# The Influence of Rice Types and Boiling Time on Glycemic Index: An In Vivo Evaluation Using the ISO 2010 Method

**DOI:** 10.3390/foods14010012

**Published:** 2024-12-25

**Authors:** Anna Vîrlan, Lidia Coșciug, Dinu Țurcanu, Rodica Siminiuc

**Affiliations:** Faculty of Food Technology, Technical University of Moldova, 168, Stefan cel Mare bd, MD-2004 Chisinau, Moldova; anna.virlan@doctorat.utm.md (A.V.); lidia.cosciug@toap.utm.md (L.C.); dinu.turcanu@adm.utm.md (D.Ț.)

**Keywords:** glycemic index (GI), rice types, boiling time, dietary glycemic control, postprandial glycemic response, personalized dietary guidance

## Abstract

Effective blood glucose management is essential for individuals with type 1 diabetes, particularly when dietary adjustments involve staple foods like rice. As a primary carbohydrate worldwide, rice significantly influences the glycemic index (GI) based on its type and cooking method. This study investigated the impact of rice type and boiling duration on the GI in healthy adults using an in vivo approach aligned with ISO 2010 standards. The glycemic response to four rice types (white round-grain, parboiled medium-grain, white long-grain, and whole-grain long-grain) was measured through postprandial blood glucose levels under both standard and extended boiling conditions to assess their implications for dietary glycemic control. Ten healthy participants (mean age 25 years, body mass index (BMI) 23.0 ± 1.6 kg/m^2^) consumed rice samples containing 50 g of available carbohydrates, prepared under controlled boiling conditions. Postprandial glycemic response was measured at regular intervals over 2 h following ingestion, with glucose solution as a reference food. The GI was calculated based on the incremental area under the glycemic response curve for each rice sample. Extended boiling significantly increased the GI across all rice types. White round-grain rice exhibited the highest relative increase (+15.8%) in the GI, while whole-grain long-grain rice, despite showing a greater percentage increase (+25.4%), maintained the lowest overall GI due to its high amylose and fiber content. Rice types with higher amylopectin content demonstrated faster glycemic responses and higher GI compared to high-amylose types. This study highlights rice type and cooking time as critical factors influencing postprandial glycemic response. Shorter boiling durations may benefit individuals requiring strict glycemic control, particularly those with diabetes, underscoring the importance of personalized dietary guidance for managing glycemic outcomes effectively.

## 1. Introduction

Diabetes mellitus is one of the primary challenges to global public health, with a continuously rising number of cases reported annually, especially in low- and middle-income countries [1,2,3]. According to the International Diabetes Federation, over 537 million people worldwide were estimated as living with diabetes in 2021, a figure projected to rise to 783 million by 2045 [4]. This trend underscores the need for effective strategies in diabetes prevention and management, focusing on nutritional interventions and dietary education.

Rice is a vital carbohydrate source and a staple food for over 4 billion people globally, playing a key role in diets across various regions [5,6,7,8]. In the agricultural year 2022/2023 alone, global rice consumption was estimated at 520.4 million metric tons [9]. Comprising mainly starch (72–75%), rice provides a rapid energy source, yet its different types—such as white, whole-grain, short-grain, medium-grain, and long-grain rice—vary significantly in amylose/amylopectin content and resulting glycemic response. Recent studies indicate that white rice, specifically those with high amylopectin content, yields a high glycemic index (GI) due to the rapid digestibility of its starch. Therefore, for individuals with diabetes, white rice consumption may lead to rapid glucose spikes, representing a notable challenge to effective diabetes management [10,11,12].

Over the past decade, research has increasingly focused on the relationship between rice consumption and glycemic response, especially in diabetic and metabolically at-risk populations. Several studies have highlighted that the amylose-to-amylopectin ratio in rice plays a critical role in determining starch digestibility and postprandial glycemic levels. Based on prior research, high-amylopectin varieties, such as white short-grain rice, are hypothesized to have a higher GI, while high-amylose varieties, such as whole-grain or parboiled rice, are expected to exhibit slower digestion and lower glycemic responses. This study aims to experimentally evaluate these effects under standardized conditions [13,14,15]. The glycemic index of rice is significantly influenced by cooking processes, highlighting their critical role in glycemic response modulation. For example, prolonged boiling enhances starch gelatinization, which increases enzymatic accessibility and accelerates glucose release into the bloodstream, while moderate boiling helps maintain a more stable glycemic response [16,17]. These findings underscore the importance of both rice type and preparation method in glycemic control, offering practical strategies for diabetes management.

GI control thus becomes essential in formulating safe, effective diets for diabetic individuals. In this context, both rice type and cooking method, especially boiling time, significantly influence the GI of rice-based foods. Extended boiling increases starch digestibility via gelatinization, which can accelerate starch conversion to glucose, leading to rapid absorption in the bloodstream. This effect positions cooking time as an important factor in glycemic response control, alongside rice selection. For example, whole-grain rice, with a higher amylose content, exhibits slower gelatinization and a lower GI, making it a preferred dietary option for stable glycemic response [7,13,14,15].

Dietary management for individuals with diabetes can be complex, as certain foods, such as those containing gluten, may need to be avoided, making rice an important alternative. However, the effects of rice consumption on glycemic response remain underexplored. Adapting the diet based on the glycemic index and preparation method can provide significant benefits for metabolic stability and risk reduction [16,17].

This study investigates the influence of boiling time on the GI of various rice types, using the ISO 26642:2010 method to determine the in vivo glycemic response in adults. By assessing how cooking time and rice type affect blood glucose, this study provides practical insight into how food choice and preparation methods can support glycemic control in metabolically vulnerable individuals. The findings aim to contribute to tailored dietary recommendations, supporting personalized diets to optimize glycemic control and a reduced risk of metabolic complications.

## 2. Materials and Methods

### 2.1. Participants

This study involved 10 participants (4 men and 6 women) aged between 20 and 35 years (mean age: 25 ± 3 years), with a body mass index (BMI) within the normal-weight range of 18.5–24.9 kg/m^2^ (mean BMI: 23.0 ± 1.6 kg/m^2^), according to World Health Organization (WHO) guidelines [18]. All participants were native residents of Chișinău, the capital of the Republic of Moldova, ensuring a homogeneous group with shared environmental and dietary influences. To mitigate individual variations in glucose metabolism, participants were carefully selected based on strict inclusion criteria, including the absence of metabolic disorders, medication use, or supplements that could affect glucose regulation.

Additionally, all participants underwent a fasting period (10–12 h) before testing to ensure standardized baseline conditions and minimize individual variability in glycemic response.

Inclusion criteria required participants to meet the specified age and BMI ranges and to have no preexisting metabolic conditions, such as type 1 or type 2 diabetes, celiac disease, or other disorders affecting glycemic response. Exclusion criteria included smoking, use of medications or supplements affecting glucose metabolism during the two weeks preceding the study, and any chronic conditions that could influence glycemic regulation.

The sample size of 10 participants was determined based on the exploratory design of this study and the logistical constraints associated with rigorous in vivo testing. To address the limitations posed by a smaller sample, strict measures were implemented to control external variables, such as participant selection criteria and standardized experimental conditions. These measures enhanced the reliability of the results and established a foundational basis for future, more comprehensive studies.

To ensure the validity of the results, all participants were nonsmokers and had not used any medications or supplements with the potential to influence glucose metabolism in the previous two weeks. This study adhered to the ethical principles outlined in the Declaration of Helsinki, ensuring full compliance with internationally recognized standards for human research ethics. All measurements were performed at the Gastroenterology Department’s laboratory at the Timofei Moșneaga Republican Clinical Hospital in Chișinău.

### 2.2. Test Samples

Four types of rice with different properties were selected for this study to facilitate a comprehensive analysis of each type’s impact on postprandial glycemic index. The selected samples include the following:

White round-grain rice (WRGR)—A variety with a softer consistency and high amylopectin content, which facilitates a faster digestion rate. It is commonly utilized in liquid-based dishes such as soups and porridges and has a higher glycemic index compared to other types.

Parboiled medium-grain white rice (PMGWR)—Having undergone a parboiling process, this medium-grain rice requires less cooking time than white round-grain rice. It also has a high amylopectin content, which may result in an intermediate glycemic response.

White long-grain rice (WLGR)—With an elongated structure and moderate amylose content, this type of rice is characterized by a lower glycemic index under standard cooking conditions. It was included to examine the effects of grain shape and starch composition on glycemic response.

Whole-grain long-grain rice (WGLGR)—This variety retains its bran and contains high levels of amylose and fiber. As a preferred choice in low-GI diets, it was selected to facilitate a comparison of the impact of fiber and amylose on glucose absorption during extended cooking.

For each rice sample, a fixed rice-to-water ratio of 1:5.7 was maintained, and the preparation method included both standard boiling as per the manufacturer’s instructions and extended boiling, with an additional 10 min beyond the recommended duration. This approach aimed to investigate how cooking time affects starch gelatinization and, consequently, the glycemic index (Table 1).

The nutritional values of the rice samples were determined based on the information indicated on product packaging. The amylose and amylopectin contents were provided by the manufacturers, while moisture content was experimentally analyzed [19] (the moisture analysis was necessary to accurately calculate the rice-to-water ratio during boiling) (Table 2). Available carbohydrates were calculated using the following formula:

Available Carbohydrates (AvCHO) = 100 g − (Moisture + Protein + Fat + Fiber)(1)

The control sample (glucose solution) was obtained by dissolving 50 g of glucose powder in 250 mL of water.

After boiling, all samples were cooled to room temperature for 10 min before being consumed by the participants. Each participant consumed 50 g of available carbohydrates (AvCHO) from rice [10].

### 2.3. Experimental Design

This study employed a randomized, controlled experimental design to evaluate the impact of different rice types and preparation methods on postprandial glycemic response in healthy participants. A total of eight rice samples were tested, comprising four rice types, each prepared using two distinct cooking methods: standard boiling (following the manufacturer’s instructions) and extended boiling (with an additional 10 min beyond the recommended time).

Each participant tested one rice sample per day, ensuring that all participants completed eight test sessions. This approach allowed for an in-depth evaluation of both the rice type and preparation method on the glycemic index (GI). To minimize potential confounding factors, such as residual or carry-over effects, a 7-day interval was maintained between test sessions, ensuring sufficient metabolic stabilization and reliable glycemic response measurements.

The randomized allocation of rice samples and cooking methods to test days ensured that variations in daily metabolic conditions among participants were evenly distributed across the experimental conditions. This design facilitated a direct comparison of the experimentally determined glycemic index (GI) values across all rice types and preparation methods under controlled conditions.

Glycemic index determination. The glycemic index of each rice type was determined following the ISO 26642:2010 standardized methodology [20], which provides guidelines for GI determination and food classification recommendations. Each participant consumed the rice sample after a fasting period of 10–12 h. Capillary blood glucose levels were measured at fasting baseline and subsequently at 15, 30, 45, 60, 90, and 120 min after beginning rice consumption. These time points enabled a comprehensive evaluation of the postprandial glycemic response. Capillary blood samples (40 µL) for each measurement were collected in a glass capillary tube, mixed with physiological saline solution (15 mL), and centrifuged for 10 min at 3000 rpm to obtain the serum required for analysis. Glucose concentration was measured using an automatic biochemical analyzer, Stat Fax^®^ 1904 (Awareness Technology, Inc., based in Palm City, FL, USA), based on the glucose oxidase method. This method involves measuring glucose concentration via a specific enzymatic reaction.

Glycemic values were obtained photometrically at a wavelength of 340 nm and a controlled temperature of 37 °C. Measurements were conducted under controlled laboratory conditions to ensure accuracy and consistency. The analyzer was regularly calibrated using standard glucose solutions, and internal control samples were analyzed to verify the reliability of the equipment and reagents.

For each type of rice, the area under the glycemic response curve (AUC) was calculated using the trapezoidal method, according to the following formula:

(2) $$A=\sum_{i=1}^{n-1}\frac{Y_{i}+Y_{i+1}}{2}\;(X_{i+1}-X_{i})$$

where:

*Y_i_* și *Y_i +1_* represent the blood glucose values at successive time points (values on the *Y*-axis), and

*X_i_* și *X_i +1_* represent the corresponding time points (values on the *X*-axis).

The glycemic index (*GI*) was calculated as the ratio between the incremental area under the postprandial glycemic curve for rice and the area under the glycemic curve obtained by consuming the glucose solution (reference), according to the following formula:

(3) $$GI=\frac{\mathrm{AUC}\;\mathrm{rice}}{\mathrm{AUC}\;\mathrm{glucose}}\times 100$$

where

AUC rice is the area under the glycemic response curve for rice;

AUC glucose is the area under the glycemic response curve for the glucose solution.

## 3. Results

### 3.1. Preprandial Blood Glucose

The mean preprandial blood glucose values before glucose consumption show minor variation across the three tests, ranging from 4.61 mmol/L to 4.69 mmol/L. The standard deviation (STDEV) is very low for all tests, indicating minimal variability between measurements, with values ranging from 0.16 to 0.19 mmol/L. The coefficient of variation (CV), which expresses relative variability, is also low, with values between 3.46% and 4.08%. The mean coefficient of variation (CV) across all three tests was 0.9%, highlighting a high level of reproducibility in preprandial blood glucose values among participants.

### 3.2. Glycemic Response to Consumption of 50 g of Glucose

The results regarding the glycemic response to the consumption of 50 g of glucose reveal notable variability both intra-individually and inter-individually (Table 3).

Intra-individual variability. At the individual level, the mean postprandial blood glucose values ranged from 6.30 to 7.01 mmol/L across the three tests. The standard deviation was elevated, indicating substantial variability in glycemic response between tests for each participant. The intra-individual coefficient of variation (CV) ranged from 20.89% to 29.39%, reflecting significant variability in the glycemic response within individuals under varying test conditions.

Inter-individual variability. In comparison, inter-individual variability was much lower. The average blood glucose among participants showed minimal variation between tests, with values ranging from 6.64 to 6.67 mmol/L. The standard deviation was lower in this case, ranging from 0.21 to 0.25 mmol/L, indicating greater consistency among participants than within the repeated tests of a single participant. The inter-individual coefficient of variation (3.13–3.79%) reflects a high level of uniformity in glycemic response at the group level, highlighting the reliability of the group averages for glucose response. Such consistency at the group level is crucial for validating the generalizability of the findings.

### 3.3. Glycemic Response to Rice Samples Consumption over Time

The glycemic response (GR) to liquid rice porridge consumption showed that cooking time has a significant effect on the GI. For WRGR, when cooked for 20 min, the average GI was 83.0. When the cooking time was extended by 50% (to 30 min), the GI increased to 96.2, representing a relative increase of 15.8%. For PWMGR, when cooked for 25 min, the average GI reached 67.0. With an additional 40% cooking time (up to 35 min), the GI rose to 74.9, an increase of 11.8%.

**Figure 1:**
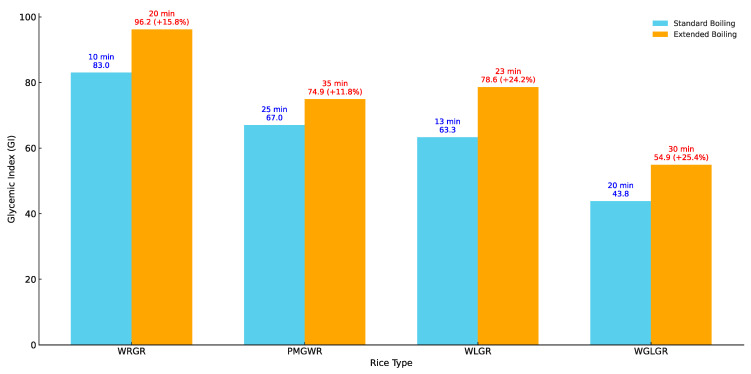
GI variation in rice samples depending on boiling time. WRGR—white round-grain rice; PWMGR—parboiled white medium-grain rice; WLGR—white long-grain rice; WGLGR—whole-grain long-grain rice.

The same pattern was observed for WLGR. When cooked for 13 min, the average GI was 63.3. When the cooking time was extended by 77% (to 23 min), the GI rose to 78.6, a difference of 24.2%. For WGLGR, cooked for 10 min, the initial GI was 43.8. Doubling the cooking time (to 20 min) increased the GI to 54.9, representing a 25.4% increase (Figure 1).

The results highlight a direct relationship between cooking duration and the increase in glycemic index for all types of rice. Longer cooking times promote greater starch gelatinization, enhancing digestibility and raising the glycemic index [21,22]. These findings provide valuable insights for managing blood glucose levels through dietary choices, particularly for individuals with diabetes. This relationship suggests that less-cooked rice may help maintain a lower glycemic index.

The trend of increasing GI with boiling time. For all tested rice types, a longer cooking time led to higher GI values, as shown in Figure 1. This trend was consistently observed across all samples, including WRGR, PWMGR, WLGR, and WGLGR. WRGR had the highest GI among all rice types, reaching peak values when cooking time was extended. PWMGR exhibited an intermediate GI, but it also increased significantly with prolonged cooking. WLGR showed lower GI values at the standard cooking time, but these rose considerably with extended boiling. WGLGR had the lowest GI among all types at the standard cooking time; however, even this type demonstrates a significant rise when the cooking time was doubled (Figure 1).

The chart illustrates the percentage differences in the GI for each type of rice across various cooking durations. Whole-grain long-grain rice (WGLGR) exhibits the highest percentage variability, reflecting its pronounced sensitivity to changes in cooking duration.

## 4. Discussions

The glycemic index (GI) is a key dietary parameter that plays a pivotal role in sharing postprandial glucose levels and overall metabolic health. Several factors, including rice variety, the amylose-to-amylopectin ratio, cooking method, and the rice-to-water ratio, influence the GI of rice. It is generally accepted that varietal effects on the GI are primarily mediated by amylose content, which influences the texture of cooked rice and is an important aspect in selecting rice for specific applications [23,24,25,26,27,28,29].

This study demonstrate that rice types and cooking duration significantly influence GI, as evidenced by the increases observed with extended cooking times. Tailoring dietary interventions, such as adjusting cooking times and selecting rice varieties, offers practical tools for glycemic management and overall metabolic health.

The influence of moisture on GI. Specifically, increased moisture reduces molecular order at the microstructural level and the relative crystallinity of rice starch. Water and heat disrupt the crystalline structure of starch, facilitating gelatinization. This process increases enzymatic accessibility and accelerates glucose release, raising the GI [22]. As starch absorbs water, glucose chains unravel and become more accessible to digestive enzymes, accelerating the conversion of starch into glucose [30,31,32,33]. It is important to acknowledge that the conclusions drawn from this study are based on a limited sample size and should be interpreted as preliminary insights. Larger and more diverse participant groups will be necessary to validate these findings and expand their applicability to broader populations, particularly those with specific dietary or metabolic needs. Nonetheless, the results offer valuable initial evidence to guide further exploration and dietary recommendations. These findings are consistent with previous research, which shows that increased moisture during rice preparation facilitates complete starch gelatinization, resulting in a higher GI. For example, Fan et al. (2024) found that starch in high-moisture rice becomes more digestible and releases glucose more rapidly into the bloodstream. Similarly, other studies have demonstrated that insufficient moisture inhibits starch expansion, limiting digestibility and maintaining a lower GI [14,28,34,35].

Influence of boiling time on GI. The results obtained in this study suggest that prolonged boiling time significantly increases the GI for all types of rice. The relationship between cooking duration and the increase in the GI was determined by systematically comparing glycemic measurements for each rice type at different boiling times, employing standardized ISO 26642:2010 methods for GI determination. This effect can be explained by the structural changes in starch, which, under prolonged heat and moisture exposure, undergoes a complete gelatinization process, leading to greater carbohydrate availability for digestion [36,37].

This effect of boiling duration on the GI is consistent with findings reported by Murillo et al. [38], which indicate that prolonged boiling increases the GI [14,38,39], while shorter cooking times result in more controlled glucose levels [40,41]. Similarly, studies by Dipnaik and Kokare (2017) highlight the role of high amylose content and fiber in reducing starch digestibility and slowing glucose absorption, findings that correspond to the lower GI values observed for WGLGR even after extended boiling [29].

Kaur et al. (2016) highlighted that rice types high in amylopectin, such as white short-grain rice, exhibit higher GI values due to rapid starch digestibility, findings consistent with our observations regarding white round-grain rice [14]. Similarly, Murillo et al. (2022) demonstrated that extended boiling increases the GI through starch gelatinization, paralleling our results for prolonged cooking across all rice types tested [38].

These comparisons underscore the contribution of our study to the broader understanding of glycemic responses to rice. By focusing on liquid-consistency preparations, this research complements previous work by providing insights into the effects of water-to-rice ratios and boiling duration, as described by Yamaguchi et al. (2021) and Fan et al. (2024) [21,22]. Collectively, these findings not only validate the robustness of the ISO 26642:2010 methodology used in this study but also emphasize the clinical significance of selecting appropriate rice types and cooking methods for glycemic management, particularly for populations with specific dietary needs, such as those with diabetes.

These results highlight the need to tailor boiling times and rice variety selection for control in individuals with metabolic conditions.

Comparison between rice types. The rice samples analyzed showed significant variations in the glycemic index (GI) based on rice type. This study compared four main rice types—white round-grain, parboiled white medium-grain, white long-grain, and whole-grain long-grain. The observed GI differences among these types are attributed to their distinct amylose and amylopectin contents, which influence starch digestibility and postprandial glycemic response [14,29,42,43].

Amylopectin-rich rice, such as white round-grain, exhibited higher glycemic index values due to rapid gelatinization, while high-amylose rice like whole-grain retained a lower GI even after prolonged boiling [44]. Amylose, a linear glucose chain, is more resistant to digestion, making this rice type preferable in dietary regimens for individuals who require stricter glycemic control. The fiber content in whole-grain rice forms a physical barrier around starch granules, delaying enzymatic access and reducing the rate of glucose release into the bloodstream [8].

These findings align with other studies investigating the effects of amylose and amylopectin content on glucose and insulin responses in different rice types, which have shown that rice with high amylose content exhibits lower initial responses and slower declines in both glucose and insulin levels [14,45,46]. These findings corroborate previous research, reinforcing the role of amylose and fiber content in modulating glycemic responses and supporting the dietary use of high-amylose rice for glycemic control.

Limitations of the study. The primary limitation of this study is its relatively small sample size, which may restrict the generalizability of the findings and reduce their statistical power. A larger number of participants would allow a more detailed assessment of glycemic response variability, thus contributing to stronger statistical validity. Additionally, although all participants were healthy and met the inclusion criteria, individual variations in glucose metabolism, factors such as genetic predispositions, prior dietary patterns, and physical activity levels, likely contributed to individual variations in glycemic responses, underscoring the need for personalized assessments in future research. Future studies with larger and more diverse samples are necessary to evaluate the extent of such variability.

Despite these limitations, this study serves as an essential first step in understanding the glycemic impact of rice and cooking methods commonly used in the Republic of Moldova, providing a foundation for further research in this area. Another limitation is the exclusive inclusion of healthy participants without metabolic conditions such as type 2 diabetes or gluten intolerance (except for participants with celiac disease). This restriction limits the applicability of the results to patient groups that would benefit most from dietary adjustments for glycemic index control. Additionally, only a few types of rice were considered in the study (such as long and short grain, white and whole-grain rice), using only one preparation method: boiling. Other varieties of rice (such as black or red rice) and cooking methods (steaming, baking) might influence digestibility and the glycemic index in a different way, and these aspects should be explored in future research.

The inclusion and exclusion criteria, though applied consistently, should be explicitly detailed in future studies to ensure clarity and enhance the replicability of findings. Future studies could benefit from integrating additional assessments, such as insulin sensitivity measurements or genetic testing, to provide a more comprehensive understanding of individual variations in glycemic responses.

Suggestions for future research. Future studies should prioritize expanding the sample size and including more diverse participant groups to enhance statistical validity and capture a broader range of glycemic responses, particularly among individuals with specific metabolic conditions. Investigating other types of rice, such as black or red varieties, as well as alternative preparation methods like steaming or baking, would provide a more comprehensive understanding of their influence on the glycemic index. Furthermore, incorporating longitudinal study designs could elucidate the cumulative effects of dietary interventions on glycemic control, metabolic health, and diabetes progression. The findings of this study have significant clinical applications, particularly in tailoring dietary guidelines for individuals with diabetes or metabolic disorders, which could be adapted to regions with dietary patterns similar to those in Moldova. Addressing these research gaps would strengthen the evidence base for dietary strategies targeting glycemic management and overall metabolic health.

## 5. Conclusions

This study underscores the significant role of rice type and cooking method in influencing the GI, offering critical insights for dietary management in individuals with diabetes or other metabolic disorders. The results demonstrate that white rice with high amylopectin content exhibits a higher GI, due to its rapid starch gelatinization and increased enzymatic digestibility, leading to a rapid and elevated glycemic response, particularly when cooked for extended periods.

These findings emphasize the significant influence of extended boiling on the glycemic index across all rice types and reinforce the critical role of rice type and cooking time in developing personalized dietary strategies for glycemic control. By addressing these aspects, this research contributes to evidence-based dietary strategies aimed at optimizing glycemic outcomes and improving overall metabolic health. 

## Figures and Tables

**Table 1:** Cooking conditions of rice samples.

Rice Sample	Rice Mass Containing 50 g CHO, g	Water	Rice/Water Ratio	BT 1 *, min	BT 2, min	Mass After Boiling, g
WRGR	64.3	394	1/5.7	10	20	462.96
PWMGR	65.8	366	1/5.7	25	35	430.10
WLGR	66.7	360	1/5.7	13	23	423.13
WGLGR	69.0	396	1/5.7	20	30	465.36

Boiling time—BT; * recommended boiling time (as indicated on the packaging).

**Table 2:** Nutrient content in rice samples.

Parameters	M.U.	WRGR	PWMGR	WLGR	WGLGR
Moisture	%	13.3	13.6	13.7	12.8
Protein	%	7	7.51	7	7.5
Fat	%	1	1	1	2.68
AvCHO	%	77.8	76	75	72.5
Amylose	%	13	20	22	26
Amylopectin	%	87	80	78	74
Dietary fiber	%	0.7	1.75	2.2	3.4
Energy value	Kcal (per 100 g)	324.2	351.31	354.86	344.12

WRGR—white round-grain rice; PWMGR—parboiled white medium-grain rice; WLGR—white long-grain rice; WGLGR—whole-grain long-grain rice.

**Table 3:** Mean blood glucose values (over 2 h) following consumption of 50 g of glucose.

**Fasting Glucose (mmol/L)**	**Postprandial Glycemic Response After Consuming 50 g of Glucose**							
	**Test 1**		**Test 2**		**Test 3**		**Average Values per 3 Tests**	
	**Mean** **± STDEV**	**CV**	**Mean** **± STDEV**	**CV**	**Mean** **± STDEV**	**CV**	**Mean** **± STDEV**	**CV**
	4.61 ± 0.16	3.46	4.69 ± 0.17	3.69	4.63 ± 0.19	4.08	4.64 ± 0.04	0.9
Intra-individual results (per participant across multiple tests)								
**Participants**	**Mean** **± STDEV**	**CV**	**Mean** **± STDEV**	**CV**	**Mean** **± STDEV**	**CV**	**Mean** **± STDEV**	**CV**
P. 1	6.31 ± 1.82	28.88	6.40 ± 1.79	28.55	6.54 ± 1.86	28.44	6.42 ± 0.18	2.88
P. 2	6.47 ± 1.33	20.53	6.56 ± 1.41	21.55	6.51 ± 1.37	21.00	6.51 ± 0.19	2.95
P. 3	6.8 ± 1.86	27.38	6.81 ± 1.81	26.26	6.83 ± 1.79	26.19	6.81 ± 0.14	2.10
P. 4	7.01 ± 1.84	26.18	6.97 ± 1.87	26.79	7.00 ± 1.85	26.50	7.00 ± 0.15	2.16
P. 5	6.64 ± 1.77	26.70	6.64 ± 1.77	26.70	6.61 ± 1.83	27.62	6.63 ± 0.16	2.42
P. 6	6.17 ± 1.81	29.31	6.40 ± 1.81	28.25	6.54 ± 1.75	26.81	6.37 ± 0.17	2.75
P. 7	6.43 ± 1.41	21.98	6.44 ± 1.39	21.63	6.37 ± 1.44	22.54	6.41 ± 0.19	3.04
P. 8	6.77 ± 1.84	26.84	6.80 ± 1.87	27.49	6.77 ± 1.84	27.24	6.78 ± 0.06	0.81
P. 9	7.01 ± 1.90	27.09	6.99 ± 1.90	27.15	6.96 ± 1.92	27.56	6.99 ± 0.07	0.96
P. 10	6.53 ± 1.86	28.03	6.57 ± 1.80	27.33	6.59 ± 1.86	28.22	6.56 ± 0.07	0.99
Inter-individual results (between participants)								
	6.62 ± 0.28	4.30	6.66 ± 0.22	3.35	6.67 ± 0.21	3.09	6.65 ± 0.23	3.51

P—participant.

## Data Availability

The original contributions presented in this study are included in the article. Further inquiries can be directed to the corresponding author.
